# {6,6′-Dieth­oxy-2,2′-[ethane-1,2-diylbis(nitrilo­methyl­idyne)]diphenolato}zinc(II) monohydrate

**DOI:** 10.1107/S1600536809010344

**Published:** 2009-03-25

**Authors:** Yong-Miao Shen, Wei Wang

**Affiliations:** a Department of Chemistry, Shaoxing University, Shaoxing 312000, People’s Republic of China; b Yancheng Institute of Technology, School of Chemical and Biological Engineering, Yancheng 224003, People’s Republic of China

## Abstract

The mol­ecule of the title compound, [Zn(C_20_H_22_N_2_O_4_)]·H_2_O, deviates from planarity with a dihedral angle between the two benzene rings is 18.3 (1)°. The four-coordinate Zn^II^ ion has a distorted square-planar coordination and is N_2_O_2_-chelated by the Schiff base ligand. The Zn^II^ ion and solvent water mol­ecule are located on a twofold rotation axis. The structure displays inter­molecular O—H⋯O hydrogen bonding.

## Related literature

For the chemical properties of Schiff bases, see: Lindoy *et al.* (1976 ▶). For *N*,*N*′-disalicylideneethyl­enediamine complexes, see: Correia *et al.* (2005 ▶); Cunningham *et al.* (2000 ▶). For bond-length data, see: Allen *et al.* (1987 ▶).
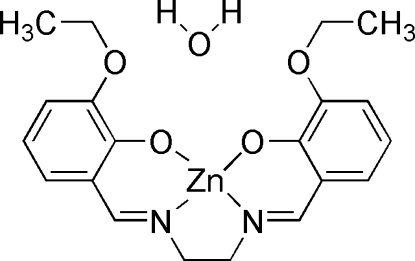


## Experimental

### 

#### Crystal data

[Zn(C_20_H_22_N_2_O_4_)]·H_2_O
*M* *_r_* = 437.78
Orthorhombic, 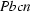

*a* = 12.6512 (16) Å
*b* = 19.986 (3) Å
*c* = 7.8708 (10) Å
*V* = 1990.1 (4) Å^3^
*Z* = 4
Mo *K*α radiation
μ = 1.27 mm^−1^
*T* = 273 K
0.25 × 0.21 × 0.17 mm

#### Data collection

Bruker APEXII CCD area-detector diffractometer
Absorption correction: multi-scan (*SADABS*; Sheldrick, 2003 ▶) *T*_min_ = 0.742, *T*_max_ = 0.813
9492 measured reflections
1855 independent reflections
1423 reflections with *I* > 2σ(*I*)
*R*_int_ = 0.031

#### Refinement

*R*[*F* ^2^ > 2σ(*F* ^2^)] = 0.034
*wR*(*F* ^2^) = 0.101
*S* = 1.04
1855 reflections
133 parameters
1 restraint
H atoms treated by a mixture of independent and constrained refinement
Δρ_max_ = 0.24 e Å^−3^
Δρ_min_ = −0.46 e Å^−3^

### 

Data collection: *APEX2* (Bruker, 2004 ▶); cell refinement: *SAINT-Plus* (Bruker, 2001 ▶); data reduction: *SAINT-Plus*; program(s) used to solve structure: *SHELXS97* (Sheldrick, 2008 ▶); program(s) used to refine structure: *SHELXL97* (Sheldrick, 2008 ▶); molecular graphics: *XP* in *SHELXTL* (Sheldrick, 2008 ▶); software used to prepare material for publication: *XP* in *SHELXTL*.

## Supplementary Material

Crystal structure: contains datablocks I, global. DOI: 10.1107/S1600536809010344/pk2159sup1.cif

Structure factors: contains datablocks I. DOI: 10.1107/S1600536809010344/pk2159Isup2.hkl

Additional supplementary materials:  crystallographic information; 3D view; checkCIF report

## Figures and Tables

**Table 1:** Hydrogen-bond geometry (Å, °)

*D*—H⋯*A*	*D*—H	H⋯*A*	*D*⋯*A*	*D*—H⋯*A*
O3—H3*A*⋯O1^i^	0.807 (10)	2.91 (5)	3.071 (4)	94 (3)

Symmetry code: (i) 
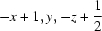
.

**Fig. 1.**
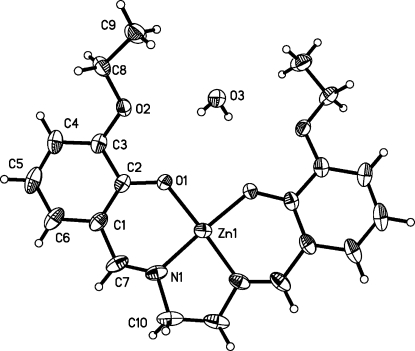
The independent molecules of the title compound, showing 30% probability displacement ellipsoids and the atom-numbering scheme.

## supplementary crystallographic information

### Comment

The schiff bases have been extensively studied as effective ligands for metal ions and used in the mechanism of many biochemical processes (Lindoy *et al.*, 1976). *N*,*N*-disalicylideneethylenediamine type schiff bases ligands present versatile steric, electronic and lipophilic properties (Correia *et al.* 2005; Cunningham *et al.* 2000). We report here the synthesis and crystal structure of the title compound. The molecular structure is shown in Fig.1. The values of the geometric parameters in the structure are normal (Allen *et al.*, 1987). The interplanar angles beween the the two phenyl group is 18.3 (1)°. The four-coordinate Zn gives plane coordination.

### Experimental

A mixture of 6,6'-Diethoxy-2,2'-(ethane-1,2-diyldiiminodimethylene)diphenol (0.1 mmol) and zinc acetate (0.1 mmol) in absolute methanol (20 ml) was heated at 50 centidegree and stirred for 30 min, then filtered. The resulting clear orange solution was moved to a tube, some ethyl ether was added, and then after 14 days, block-shaped crystals of the title complex suitable for X-ray diffraction analysis were obtained(yield: about 40%).

### Refinement

The H atoms were fixed geometrically and were treated as riding on their parent C atoms, with C–H distances in the range of 0.93–0.97Å and with *U*_iso_(H) = 1.2*U*_eq_(parent atom), or *U*_iso_(H) = 1.5*U*_eq_(C_methyl_). The coordinates of the water H atom were found in a difference Fourier map and refined with *U*_iso_(H) = 1.2*_U_*_eq_(O).

### Crystal data

**Table:**

[Zn(C_20_H_22_N_2_O_4_)]·H_2_O	*F*(000) = 912
*M**_r_* = 437.78	*D*_x_ = 1.461 Mg m^−^^3^
Orthorhombic, *P**b**c**n*	Mo *K*α radiation, λ = 0.71073 Å
Hall symbol: -P 2n 2ab	Cell parameters from 2488 reflections
*a* = 12.6512 (16) Å	θ = 3.3–24.0°
*b* = 19.986 (3) Å	µ = 1.27 mm^−^^1^
*c* = 7.8708 (10) Å	*T* = 273 K
*V* = 1990.1 (4) Å^3^	Needle, colourless
*Z* = 4	0.25 × 0.21 × 0.17 mm

### Data collection

**Table:**

Bruker APEXII CCD area-detector diffractometer	1855 independent reflections
Radiation source: fine-focus sealed tube	1423 reflections with *I* > 2σ(*I*)
graphite	*R*_int_ = 0.031
φ and ω scans	θ_max_ = 25.5°, θ_min_ = 1.9°
Absorption correction: multi-scan (*SADABS*; Sheldrick, 2003)	*h* = −15→13
*T*_min_ = 0.742, *T*_max_ = 0.813	*k* = −24→23
9492 measured reflections	*l* = −9→9

### Refinement

**Table:**

Refinement on *F*^2^	Primary atom site location: structure-invariant direct methods
Least-squares matrix: full	Secondary atom site location: difference Fourier map
*R*[*F*^2^ > 2σ(*F*^2^)] = 0.034	Hydrogen site location: inferred from neighbouring sites
*wR*(*F*^2^) = 0.101	H atoms treated by a mixture of independent and constrained refinement
*S* = 1.04	*w* = 1/[σ^2^(*F*_o_^2^) + (0.0592*P*)^2^ + 0.4077*P*] where *P* = (*F*_o_^2^ + 2*F*_c_^2^)/3
1855 reflections	(Δ/σ)_max_ = 0.034
133 parameters	Δρ_max_ = 0.24 e Å^−^^3^
1 restraint	Δρ_min_ = −0.46 e Å^−^^3^

### Special details

**Table:**

Geometry. All e.s.d.'s (except the e.s.d. in the dihedral angle between two l.s. planes) are estimated using the full covariance matrix. The cell e.s.d.'s are taken into account individually in the estimation of e.s.d.'s in distances, angles and torsion angles; correlations between e.s.d.'s in cell parameters are only used when they are defined by crystal symmetry. An approximate (isotropic) treatment of cell e.s.d.'s is used for estimating e.s.d.'s involving l.s. planes.
Refinement. Refinement of *F*^2^ against ALL reflections. The weighted *R*-factor *wR* and goodness of fit *S* are based on *F*^2^, conventional *R*-factors *R* are based on *F*, with *F* set to zero for negative *F*^2^. The threshold expression of *F*^2^ > σ(*F*^2^) is used only for calculating *R*-factors(gt) *etc*. and is not relevant to the choice of reflections for refinement. *R*-factors based on *F*^2^ are statistically about twice as large as those based on *F*, and *R*- factors based on ALL data will be even larger.

### Fractional atomic coordinates and isotropic or equivalent isotropic displacement parameters (Å^2^)

**Table:**

	*x*	*y*	*z*	*U*_iso_*/*U*_eq_	
Zn1	0.5000	0.475277 (19)	0.2500	0.04314 (18)	
O1	0.40642 (12)	0.40659 (8)	0.1698 (2)	0.0441 (4)	
O2	0.31927 (13)	0.29902 (8)	0.0342 (2)	0.0491 (4)	
O3	0.5000	0.26838 (19)	0.2500	0.0940 (13)	
N1	0.40920 (19)	0.54832 (10)	0.1725 (3)	0.0519 (6)	
C1	0.2641 (2)	0.47896 (14)	0.0770 (3)	0.0530 (7)	
C2	0.31464 (18)	0.41541 (12)	0.0957 (3)	0.0422 (6)	
C3	0.26222 (19)	0.35776 (13)	0.0260 (3)	0.0468 (6)	
C4	0.1632 (2)	0.36235 (17)	−0.0434 (4)	0.0619 (8)	
H4	0.1294	0.3244	−0.0849	0.074*	
C5	0.1133 (2)	0.4253 (2)	−0.0514 (4)	0.0800 (10)	
H5	0.0453	0.4284	−0.0957	0.096*	
C6	0.1628 (3)	0.48188 (19)	0.0045 (4)	0.0740 (10)	
H6	0.1289	0.5230	−0.0055	0.089*	
C7	0.3159 (2)	0.54153 (14)	0.1154 (4)	0.0588 (8)	
H7	0.2774	0.5805	0.0967	0.071*	
C8	0.2840 (2)	0.24216 (14)	−0.0638 (4)	0.0590 (8)	
H8A	0.2683	0.2559	−0.1792	0.071*	
H8B	0.2201	0.2238	−0.0142	0.071*	
C9	0.3688 (3)	0.19045 (15)	−0.0647 (4)	0.0726 (9)	
H9A	0.4320	0.2090	−0.1130	0.109*	
H9B	0.3463	0.1528	−0.1312	0.109*	
H9C	0.3827	0.1762	0.0496	0.109*	
C10	0.4568 (3)	0.61556 (13)	0.1843 (4)	0.0651 (8)	
H10A	0.4030	0.6479	0.2152	0.078*	
H10B	0.4857	0.6283	0.0748	0.078*	
H3A	0.487 (4)	0.2934 (18)	0.172 (4)	0.126 (18)*	

### Atomic displacement parameters (Å^2^)

**Table:**

	*U*^11^	*U*^22^	*U*^33^	*U*^12^	*U*^13^	*U*^23^
Zn1	0.0450 (3)	0.0357 (3)	0.0487 (3)	0.000	0.00991 (18)	0.000
O1	0.0357 (9)	0.0407 (9)	0.0558 (11)	0.0034 (7)	−0.0043 (8)	0.0012 (8)
O2	0.0455 (10)	0.0505 (10)	0.0513 (10)	−0.0085 (8)	−0.0114 (8)	0.0028 (8)
O3	0.096 (3)	0.054 (2)	0.132 (4)	0.000	−0.064 (3)	0.000
N1	0.0604 (15)	0.0394 (11)	0.0558 (14)	0.0128 (11)	0.0222 (12)	0.0062 (11)
C1	0.0456 (15)	0.0639 (18)	0.0496 (16)	0.0188 (12)	0.0072 (13)	0.0064 (13)
C2	0.0342 (12)	0.0563 (15)	0.0361 (13)	0.0052 (11)	0.0062 (10)	0.0073 (11)
C3	0.0378 (14)	0.0650 (17)	0.0376 (13)	0.0001 (12)	0.0031 (10)	0.0109 (12)
C4	0.0378 (15)	0.095 (2)	0.0526 (17)	−0.0027 (15)	−0.0049 (12)	0.0055 (16)
C5	0.0401 (17)	0.126 (3)	0.074 (2)	0.0183 (19)	−0.0085 (15)	0.012 (2)
C6	0.054 (2)	0.091 (2)	0.077 (2)	0.0321 (17)	−0.0010 (15)	0.011 (2)
C7	0.0645 (19)	0.0545 (17)	0.0575 (17)	0.0282 (15)	0.0164 (15)	0.0095 (14)
C8	0.0666 (18)	0.0623 (18)	0.0481 (15)	−0.0252 (15)	−0.0096 (14)	0.0051 (13)
C9	0.092 (2)	0.0575 (18)	0.068 (2)	−0.0142 (17)	−0.0122 (18)	−0.0091 (15)
C10	0.090 (2)	0.0338 (14)	0.071 (2)	0.0088 (13)	0.0375 (16)	0.0048 (13)

### Geometric parameters (Å, °)

**Table:**

Zn1—O1	1.9195 (16)	C4—C5	1.410 (5)
Zn1—O1^i^	1.9195 (16)	C4—H4	0.9300
Zn1—N1^i^	1.955 (2)	C5—C6	1.365 (5)
Zn1—N1	1.955 (2)	C5—H5	0.9300
O1—C2	1.311 (3)	C6—H6	0.9300
O2—C3	1.380 (3)	C7—H7	0.9300
O2—C8	1.444 (3)	C8—C9	1.489 (4)
O3—H3A	0.807 (10)	C8—H8A	0.9700
N1—C7	1.270 (4)	C8—H8B	0.9700
N1—C10	1.476 (3)	C9—H9A	0.9600
C1—C6	1.404 (4)	C9—H9B	0.9600
C1—C2	1.429 (3)	C9—H9C	0.9600
C1—C7	1.444 (4)	C10—C10^i^	1.505 (7)
C2—C3	1.438 (3)	C10—H10A	0.9700
C3—C4	1.369 (4)	C10—H10B	0.9700
			
O1—Zn1—O1^i^	88.69 (9)	C4—C5—H5	119.4
O1—Zn1—N1^i^	177.35 (8)	C5—C6—C1	121.0 (3)
O1^i^—Zn1—N1^i^	93.95 (9)	C5—C6—H6	119.5
O1—Zn1—N1	93.95 (9)	C1—C6—H6	119.5
O1^i^—Zn1—N1	177.35 (8)	N1—C7—C1	126.1 (2)
N1^i^—Zn1—N1	83.40 (15)	N1—C7—H7	117.0
C2—O1—Zn1	126.60 (15)	C1—C7—H7	117.0
C3—O2—C8	118.9 (2)	O2—C8—C9	109.0 (2)
C7—N1—C10	119.9 (2)	O2—C8—H8A	109.9
C7—N1—Zn1	125.21 (19)	C9—C8—H8A	109.9
C10—N1—Zn1	114.9 (2)	O2—C8—H8B	109.9
C6—C1—C2	119.1 (3)	C9—C8—H8B	109.9
C6—C1—C7	117.6 (3)	H8A—C8—H8B	108.3
C2—C1—C7	123.0 (3)	C8—C9—H9A	109.5
O1—C2—C1	124.1 (2)	C8—C9—H9B	109.5
O1—C2—C3	118.0 (2)	H9A—C9—H9B	109.5
C1—C2—C3	117.8 (2)	C8—C9—H9C	109.5
C4—C3—O2	123.6 (3)	H9A—C9—H9C	109.5
C4—C3—C2	121.3 (2)	H9B—C9—H9C	109.5
O2—C3—C2	115.0 (2)	N1—C10—C10^i^	109.85 (18)
C3—C4—C5	119.2 (3)	N1—C10—H10A	109.7
C3—C4—H4	120.4	C10^i^—C10—H10A	109.7
C5—C4—H4	120.4	N1—C10—H10B	109.7
C6—C5—C4	121.3 (3)	C10^i^—C10—H10B	109.7
C6—C5—H5	119.4	H10A—C10—H10B	108.2

Symmetry codes: (i) −*x*+1, *y*, −*z*+1/2.

### Hydrogen-bond geometry (Å, °)

**Table:**

*D*—H···*A*	*D*—H	H···*A*	*D*···*A*	*D*—H···*A*
O3—H3A···O1^i^	0.81 (1)	2.91 (5)	3.071 (4)	94 (3)

Symmetry codes: (i) −*x*+1, *y*, −*z*+1/2.
